# Biofilm Formation among Clinical and Food Isolates of* Listeria monocytogenes*

**DOI:** 10.1155/2013/524975

**Published:** 2013-12-29

**Authors:** Joana Barbosa, Sandra Borges, Ruth Camilo, Rui Magalhães, Vânia Ferreira, Isabel Santos, Joana Silva, Gonçalo Almeida, Paula Teixeira

**Affiliations:** Centro de Biotecnologia e Química Fina (CBQF), Laboratório Associado, Escola Superior de Biotecnologia, Universidade Católica Portuguesa/Porto, Rua Dr. António Bernardino Almeida, 4200-072 Porto, Portugal

## Abstract

*Objective*. A total of 725 *Listeria monocytogenes* isolates, 607 from various foods and 118 from clinical cases of listeriosis, were investigated concerning their ability to form biofilms, at 4°C during 5 days and at 37°C during 24 h. *Methods*. Biofilm production was carried out on polystyrene tissue culture plates. Five *L. monocytogenes* isolates were tested for biofilm formation after being exposed to acidic and osmotic stress conditions. *Results*. Significant differences (*P* < 0.01) between clinical and food isolates were observed. At 37°C for 24 h, most food isolates were classified as weak or moderate biofilm formers whereas all the clinical isolates were biofilm producers, although the majority were weak. At 4°C during 5 days, 65 and 59% isolates, from food and clinical cases, respectively, were classified as weak. After both sublethal stresses, at 37°C just one of the five isolates tested was shown to be more sensitive to subsequent acidic exposure. However, at 4°C both stresses did not confer either sensitivity or resistance. *Conclusions*. Significant differences between isolates origin, temperature, and sublethal acidic stress were observed concerning the ability to form biofilms. Strain, origin, and environmental conditions can determine the level of biofilm production by *L. monocytogenes* isolates.

## 1. Introduction

*Listeria monocytogenes* has been responsible for several outbreaks of foodborne diseases, worldwide. Listeriosis is largely confined to its risk groups of pregnant women, the elderly and immunocompromised individuals with high morbidity and mortality rates [1]. According to the European Food Safety Authority this bacterium remains a concern; the number of listeriosis cases in humans increased by 19.1% compared to 2008, with 1,645 confirmed cases recorded in 2009 [2].

*L. monocytogenes* can colonize most of the surfaces and equipment encountered in the food industry including refrigerated environments, and persistent strains have been reported [3–5]. During processing this organism can easily contaminate the final food product. Many bacteria are able to attach and colonize environmental surfaces by producing biofilms, a three-dimensional matrix of extracellular polymeric substances (EPS) [6]. Biofilms produced by *L. monocytogenes* are structurally simple in comparison to those by other organisms, and a mature biofilm community can be established after 24 h [6, 7]. Once established and in comparison with planktonic cells, biofilms have greater resistance to antimicrobial agents, to U.V. light, to desiccation, and to treatments with sanitizing agents [8, 9]. *L. monocytogenes* has been reported as capable of attaching and developing biofilms on a variety of surfaces, for example, stainless steel, polymers, and rubber gaskets [6, 8, 10]. This capacity varies depending on several factors: the strains considered [8, 11, 12], the topology of surface [13–15], the growth phase [9], the temperature [9], the growth media [16], and the presence of other microorganisms [17].

Djordjevic et al. [18] reported that apparently there is a relationship between phylogeny and the ability to produce biofilms. Environmental stress such as starvation also influences both attachment and biofilm development in *L. monocytogenes* [19, 20]; generally the ability to produce biofilms is enhanced after environmental stress exposure. It is therefore crucial to study the factors that contribute to production/variation in biofilm formation by *L. monocytogenes* strains in order to optimize preventative measures and thereby minimize the risk that biofilm production by *L. monocytogenes* presents to food industries.

The aim of this study was to characterize 725 *L. monocytogenes* isolates, 607 from various food products and 118 clinical isolates, with respect to their ability to form biofilms in 96 wells microtiter plates, at 4°C during 5 days and at 37°C during 24 h. The behavior of five food *L. monocytogenes* isolates on their ability to produce biofilms, after exposure to acidic and osmotic sublethal stresses, was also investigated.

## 2. Material and Methods

### 2.1. Origin of Isolates

A total of 725 *L. monocytogenes* isolates were studied; 607 recovered from foods by quality control Portuguese laboratories (23% serogroup IIa, 23% serogroup IIb, 9% serogroup IIc, and 85% serogroup IVb) and 118 isolates (12% serogroup IIa, 21% serogroup IIb, and 85% serogroup IVb) obtained from clinical cases of listeriosis that occurred in Portugal and collected from major Portuguese hospitals, between 2003 and 2008. These isolates were deposited and stored at −80°C in Tryptone Soya Broth supplemented with 0.6% (w/v) of yeast extract (TSBYE, Pronadisa, Madrid, Spain) containing 30% (v/v) glycerol in the *Listeria* culture collection of CBQF-Escola Superior de Biotecnologia (Porto, Portugal) and used in the current investigation.

### 2.2. Growth and Storage Conditions

Working cultures were inoculated from frozen stocks onto Tryptone Soya Agar containing 0.6% (w/w) of yeast extract (TSAYE; Pronadisa) and incubated at 37°C during 24 h.

Each strain was subcultured overnight in TSBYE and was further inoculated (10% v/v) into 10 mL of TSBYE and incubated at 37°C for 18 to 20 h. This procedure was repeated twice.

### 2.3. Biofilm Production

Biofilm production was carried out as previously described by Cerca et al. [21]. Although polystyrene is infrequently present in food production or clinical settings, it was used for practical reasons due to the high number of isolates being investigated. Each well of (Brand, Wertheim, Germany) was filled with 180 *μ*L of TSBYE and 20 *μ*L of an overnight culture obtained as described above. The plates were covered and incubated aerobically at 37°C during 24 h and at 4°C during 5 days. The biofilms were visualized with a 2% crystal violet solution and quantified by measuring the optical density (OD) at 655 nm using a plate reader (Microplate reader, Bio-Rad, Hercules, CA, USA). For classification of isolates according to their ability to form biofilms, a cut-off value was obtained. The cut-off value (ODc) for determining a biofilm producer and the classification of the isolates as nonbiofilm producers (OD ≤ ODc), weak biofilm producers (ODc < OD ≤ 2 × ODc), moderate biofilm producers (2 × ODc < OD ≤ 4 × ODc), and strong biofilm producers (4 × ODc > OD). Therefore, the isolates were classified as nonbiofilm producers, weak, moderate, or strong biofilm producers for each assay [14]. For each strain, all the experiments were performed at least six times: three wells in two different polystyrene tissue culture plates. The wells with medium and without inoculating the bacteria were used as negative controls.

### 2.4. Biofilm Formation after Exposure to Acidic and Osmotic Stresses

#### 2.4.1. Isolates and Growth

Five isolates were chosen from different plants: 1079 (serotype 1/2b-3b), 1055/4 (serotype 4b-4d-4e), 1509/2 (serotype 1/2c-3c), 1592/2 (serotype 1/2b-3b), and 1743 (serotype 4b-4d-4e, resident strain). Since these dairy isolates are commonly in contact with a wide range of environmental stresses, such as high salt concentration, low pH, and a_w_, these isolates were selected to study the effect of such stresses on biofilm formation ability.

Cultures were produced as described previously, but only 0.1 mL of the last inoculum was transferred to 10 mL of TSBYE (1 : 100) and further incubated at 37°C for 18–20 h. Each isolate was harvested by centrifugation (8877 ×g, 10 minutes, 4°C; Rotina 35R, Hettich, Germany), resuspended in 10 mL of sterile quarter strength Ringer's solution (Lab M, Lancashire, UK) and mixed to obtain an inoculum of approximately 10^7^ CFU/mL, quantified by the drop count technique [22] on TSAYE and further incubated for 24 h at 37°C.

#### 2.4.2. Biofilm Assay after Exposure to Sublethal Stresses

The sublethal conditions were previously established [23]. The inoculum prepared as described previously was inoculated (0.5 mL) into glass flasks containing 49.5 mL of BPW (Buffered Peptone Water, Lab M).

The pH and the NaCl concentration were adjusted accordingly (BPW at pH 3.5 with lactic acid (1 M, José M. Vaz Pereira, Lda, Lisbon, Portugal)) and BPW containing saturated solutions of 30%, only for isolates 1592/2 and 1743, or 40% (w/v) of NaCl (Panreac, Barcelona, Spain); cells were subjected to stress conditions for 1 h at 37°C. Samples were taken at time 0 (time of inoculation) and after 60 minutes. For each sublethal stress, a control was performed (BPW at pH = 7.0 and no added salt). The survivors were enumerated, in duplicate by the drop count technique [22] on TSAYE, and further incubated for 24 h at 37°C. The results were expressed in CFU/mL.

After the exposure to these sublethal stresses, each suspension was harvested by centrifugation (8877 ×g, 10 minutes, 4°C; Rotina 35 R) and the pellet resuspended with 50 mL of TSBYE. From this suspension, 20 *μ*L were added to three wells of sterile polystyrene tissue culture plates containing 180 *μ*L of TSBYE. The plates were covered and incubated aerobically for 24 h at 37°C and 5 days at 4°C. The quantification of biofilms was done as described above.

### 2.5. Statistical Analysis

To test significant differences between the two temperatures used and within replicates as well as between food and clinical isolates, the ANOVA test was applied using the software KaleidaGraph 4.0 (Synergy Software Reading, PA, USA).

## 3. Results and Discussion

**Figure 1:**
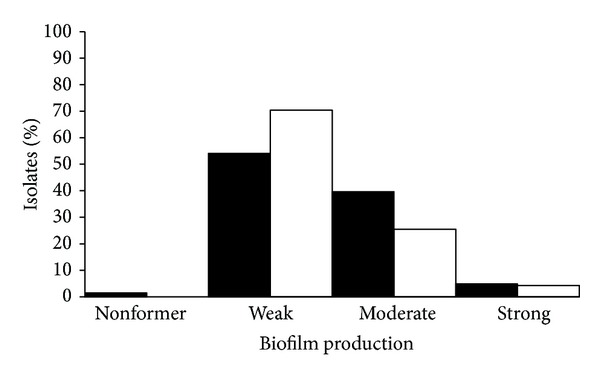
Biofilm production by clinical (□) and food (■) isolates of *L. monocytogenes* at 37°C during 24 h.

It is commonly accepted that cells in biofilms are more resistant to biocides, antibiotics, antibodies, and surfactants than are planktonic cells. Therefore, knowledge on biofilm capacity of foodborne pathogens is of major importance for the food industry, in order to define the most effective cleaning and disinfection strategies, and also in clinical settings when establishing the most appropriate therapeutic regimes. Several *L. monocytogenes* isolates from food and clinical origin were studied concerning their ability to produce biofilms at 4 and 37°C. Significant differences (*P* < 0.01) between clinical and food isolates were observed in both conditions. At 37°C for 24 h, most food isolates were classified as weak (*n* = 328; 54%), or moderate biofilm formers (*n* = 240, 40%). All clinical isolates were biofilm producers, although the majority were weak biofilm producers (*n* = 83; 70%) (Figure 1).

The percentage of food isolates that were moderate biofilm producers was slightly higher than the percentage obtained for clinical isolates at 37°C (Figure 1).

**Figure 2:**
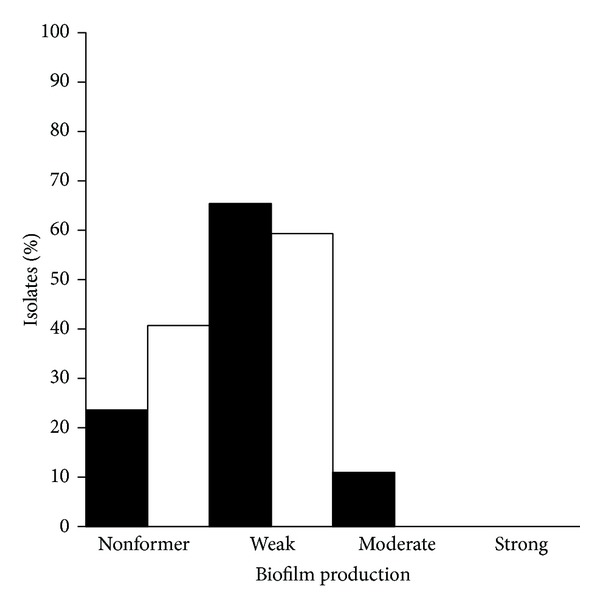
Biofilm production by clinical (□) and food (■) isolates of *L. monocytogenes* at 4°C during 5 days.

At 4°C, clinical isolates were weak (*n* = 70; 59%) or nonbiofilm producers (*n* = 48; 41%). Food isolates were non-formers (*n* = 143, 24%), weak (*n* = 397; 65%) or moderate (*n* = 67; 11%) biofilm producers (Figure 2).

Serogroups IIa and IIb (and IIc for clinical isolates) included the highest percentage of isolates showing the strongest activity to form biofilms at 37°C during 24 h; the opposite was observed for serogroup IVb (Table 1). At 4°C during 5 days, as most of the isolates were classified as non- or weak-biofilm formers, no correlation between biofilm forming capacity and serogroup was observed (Table 1).

**Figure 3:**
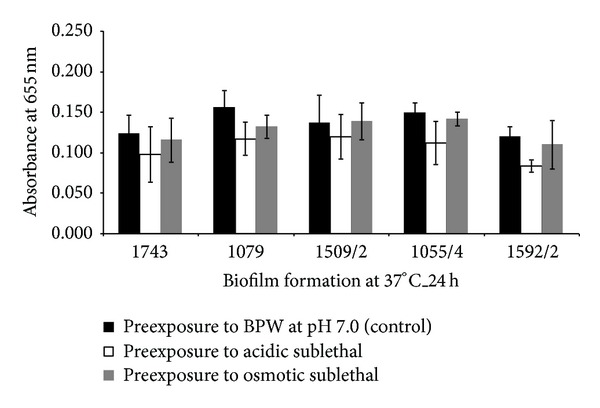
Values of absorbance at 655 nm obtained for five *L. monocytogenes* isolates after being exposed to acidic and osmotic sublethal stresses and tested for biofilm formation at 37°C during 24 h.

**Figure 4:**
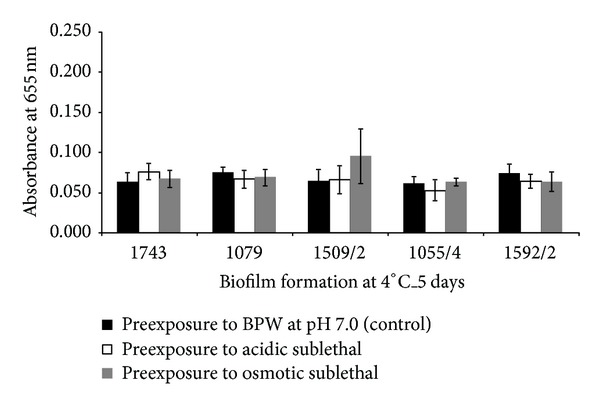
Values of absorbance at 655 nm obtained for five *L. monocytogenes* isolates after being exposed to acidic and osmotic sublethal stresses and tested for biofilm formation at 4°C during 5 days.

Five food isolates of *L. monocytogenes* were chosen in order to study the effect of two sublethal stress conditions (acidic and osmotic) in their ability to subsequently form biofilms at 37 and 4°C. After exposure to the stress conditions, it was observed that isolate 1592/2 was sensitised by acidic exposure, since its biofilm formation ability at 37°C was reduced (Figure 3). However, at 4°C, the exposure to the stress conditions neither conferred sensitivity nor resistance to all the studied isolates since no significant differences were demonstrated (Figure 4).

The influence of temperature on the ability of *L. monocytogenes* isolates to form biofilms has been reported by several authors [17, 24–26]. Chavant et al. [24] showed that *L. monocytogenes* LO28 colonized a polytetrafluoroethylene (PTFE) surface at 37°C, but not at 8°C. Di Bonaventura et al. [25] demonstrated that biofilm production on polystyrene surfaces by 44 different isolates of *L. monocytogenes* was significantly higher at 37°C than at 4°C. Norwood and Gilmour [17], however, reported two *L. monocytogenes *isolates that adhered equally at 4°C and 30°C. In the present study the temperature affected the capacity of the tested isolates to form biofilms. This capacity was shown to be dependent on the strain and on the origin of the isolate. It is important to underline the results obtained in this study in terms of strong biofilm formation by clinical isolates. Though there is a lack of literature referring to differences in biofilm production between food and clinical isolates, clinical isolates may be more adapted to temperatures close to body temperature, and this could be a possible reason for their moderate or strong biofilm production at 37°C.

Serogroups IIa and IIb (and IIc for food isolates) included the highest percentage of isolates showing the strongest activity to form biofilms at 37°C. Nilsson et al. [27] reported that among food and clinical isolates of *L. monocytogenes* (*n* = 95), serotype 1/2a (belonging to serogroup IIa) isolates produced significantly more biofilm than the other serotypes tested.

The behaviors of five food isolates of *L. monocytogenes* were investigated for their ability to produce biofilms, after exposure to acidic and osmotic sublethal stress conditions. It is reported that sublethal conditions frequently enhance the resistance of the microorganisms to subsequent stresses [28]. The cross-resistance of adapted cells to other stresses has important implications for the food industry, particularly since foods commonly encounter sublethal acidic treatments during processing [29]. Concerning strain 1592/2, after exposure to acidic sublethal stress conditions, its ability to form a biofilm at 37°C was reduced. Concerning the osmotic exposure at 37°C as well as the exposure to both sublethal conditions no differences in the capacity to form biofilm were observed at 4°C. Adrião et al. [30] investigated the behavior of some *L. monocytogenes *isolates isolated from the environment of artisanal cheese-making dairies in response to acid and salt stress. It was demonstrated that for some of these isolates, salt or acid adaptation may enhance the survival/resistance of sessile cells exposed to hypochlorite disinfection. Also Longhi et al. [15] studied a protease treatment and found that the treatment of *L. monocytogenes* with sublethal concentrations of an extracellular metalloprotease reduced the ability to form biofilms. Nilsson et al. [27] suggested that environmental conditions determine the level of biofilm production by *L. monocytogenes* isolates, independent of the rate of planktonic growth.

## 4. Conclusions

In the present work, significant differences between clinical and food isolates were observed concerning their ability to form biofilms. This ability was also influenced by the temperature used, being the biofilm formation increased at 37°C. Considering the sublethal acidic stress, biofilm formation ability was reduced only for one isolate. For sublethal osmotic stress, no changes on biofilm formation ability were observed. To explain the differences in biofilm production between food and clinical *L. monocytogenes* isolates, as well as the influence of environmental factors such as temperature, further investigations would be required, such as testing biofilm production on different surfaces relevant to food and clinical environments and the resistance of *L. monocytogenes* isolates, both in suspension and as biofilms, to sanitizing agents used in both clinical and food processing environments. Also further studies of the effect of more sublethal stresses on the behaviour of clinical and food isolates would be important.

## Figures and Tables

**Table 1:** Classification of food and clinical isolates belonging to different serogroups isolates concerning their ability to form biofilm during five days at 4°C and 24 hours at 37°C (results are expressed as % of isolates).

Serogroup	NF-C	NF-F	WF-C	WF-F	MF-C	MF-F	SF-C	SF-F
	4°C/5 days							
IIa	8	16	92	72	0	12	0	0
IIb	48	24	52	62	0	14	0	0
IIc	—	20	—	71	—	9	—	
IVb	44	28	56	63	0	9	0	0

	37°C/24 hours							
IIa	0	0	8	50	92	48	0	1
IIb	0	0	38	57	43	40	19	3
IIc	—	2	—	66	—	30	—	2
IVb	0	2	87	81	12	17	1	0

NF-C: non formers of clinical origin; NF-F: non formers of food origin; WF-C: weak formers of clinical origin; WF-F: weak formers of food origin; MF-C: moderate formers of clinical origin; MF-F: moderate formers of food origin; SF-C: strong formers of clinical origin; SF-F: strong formers of food origin.
